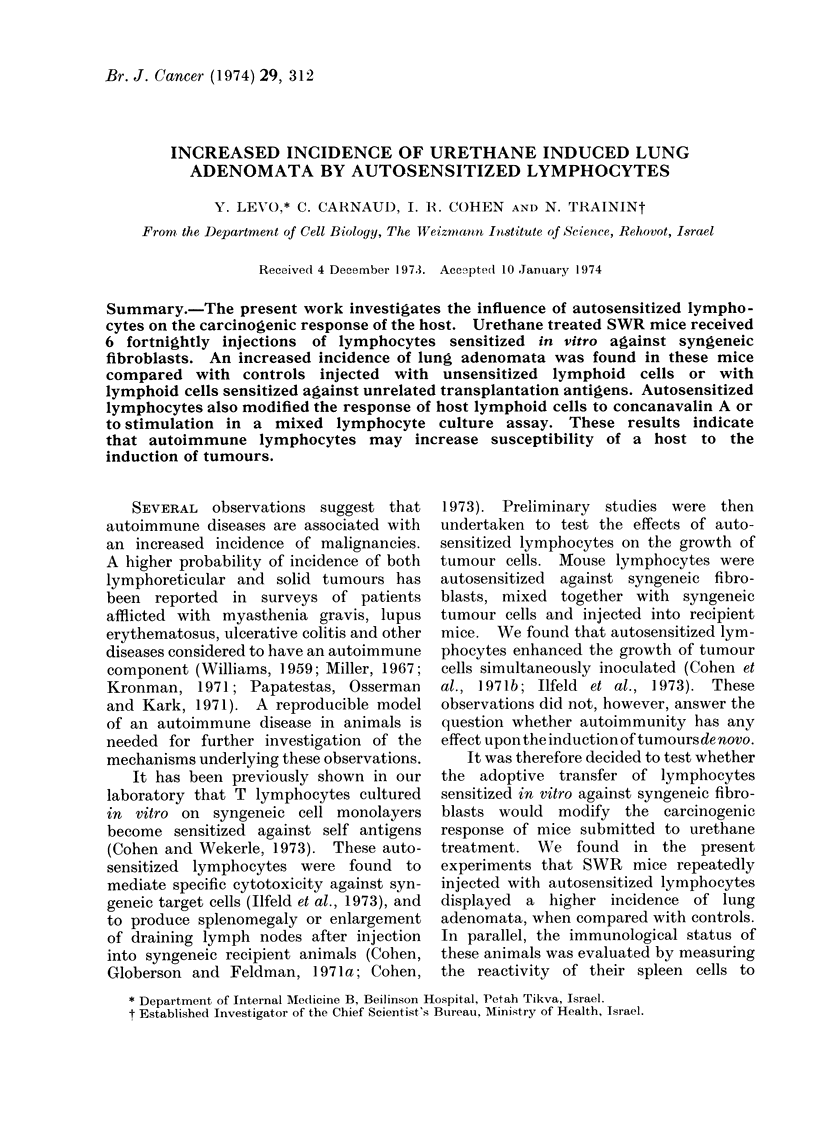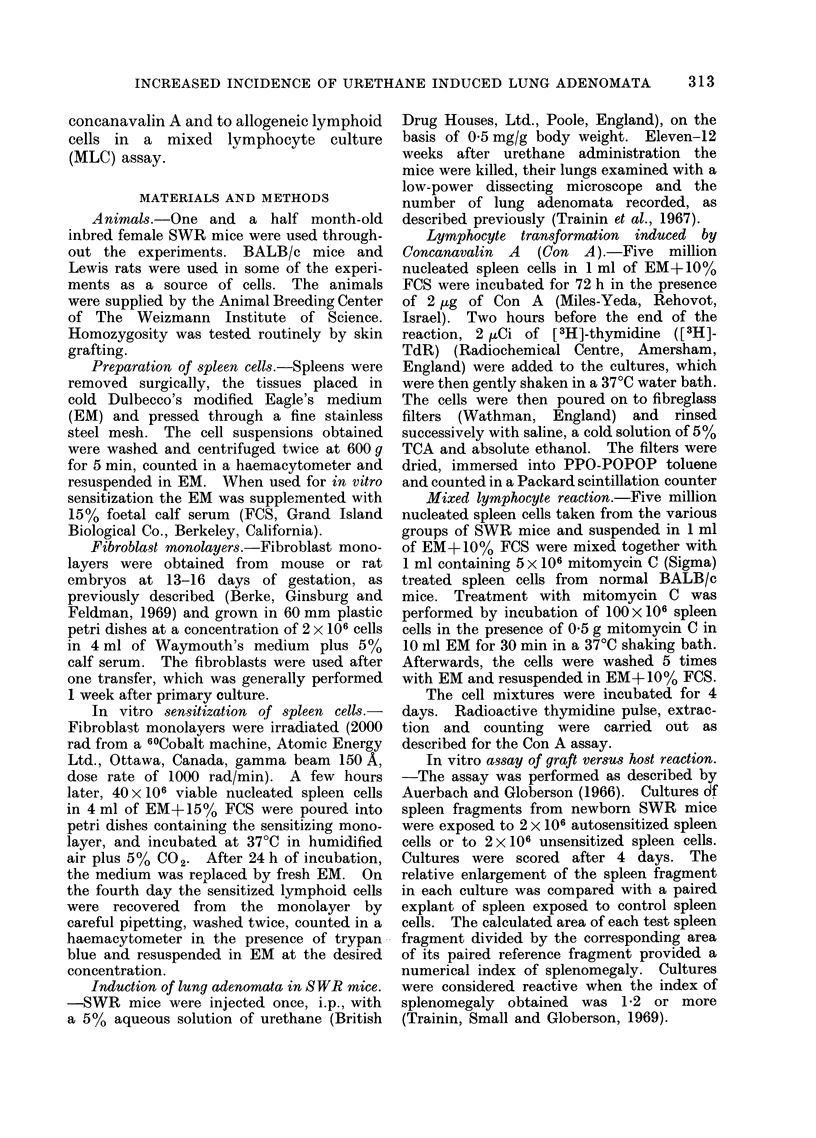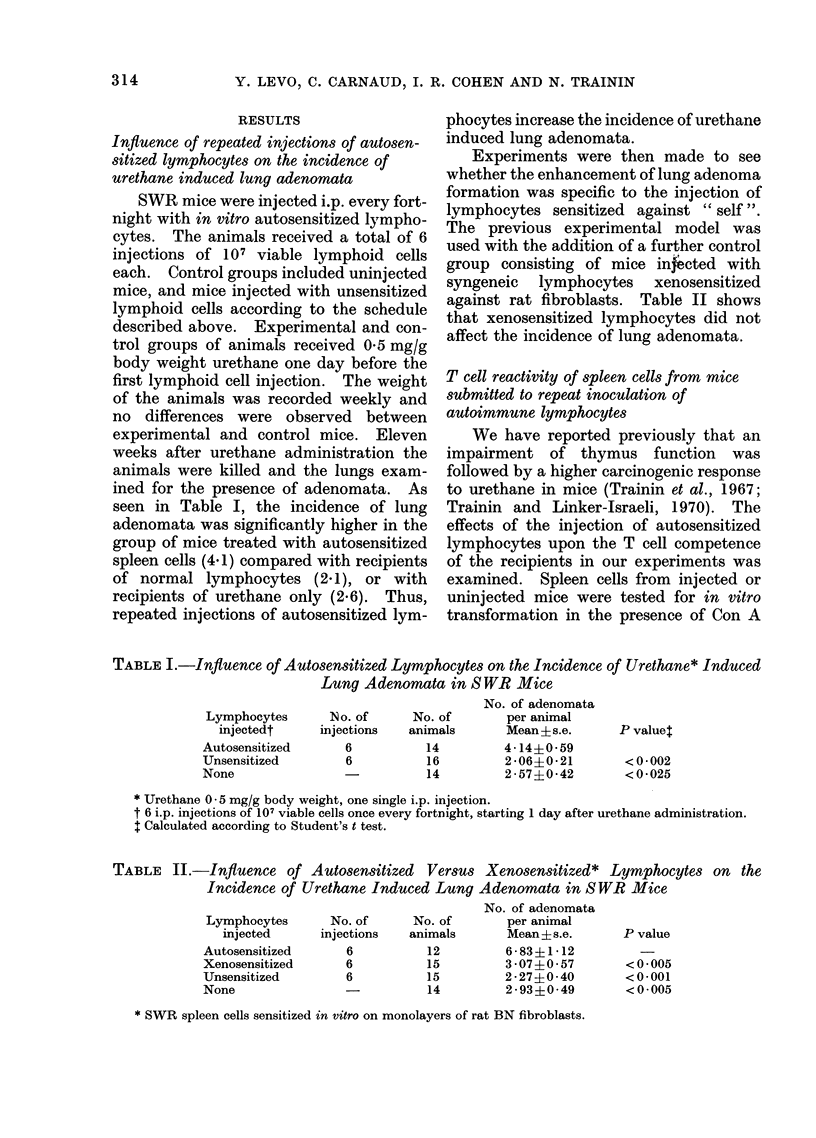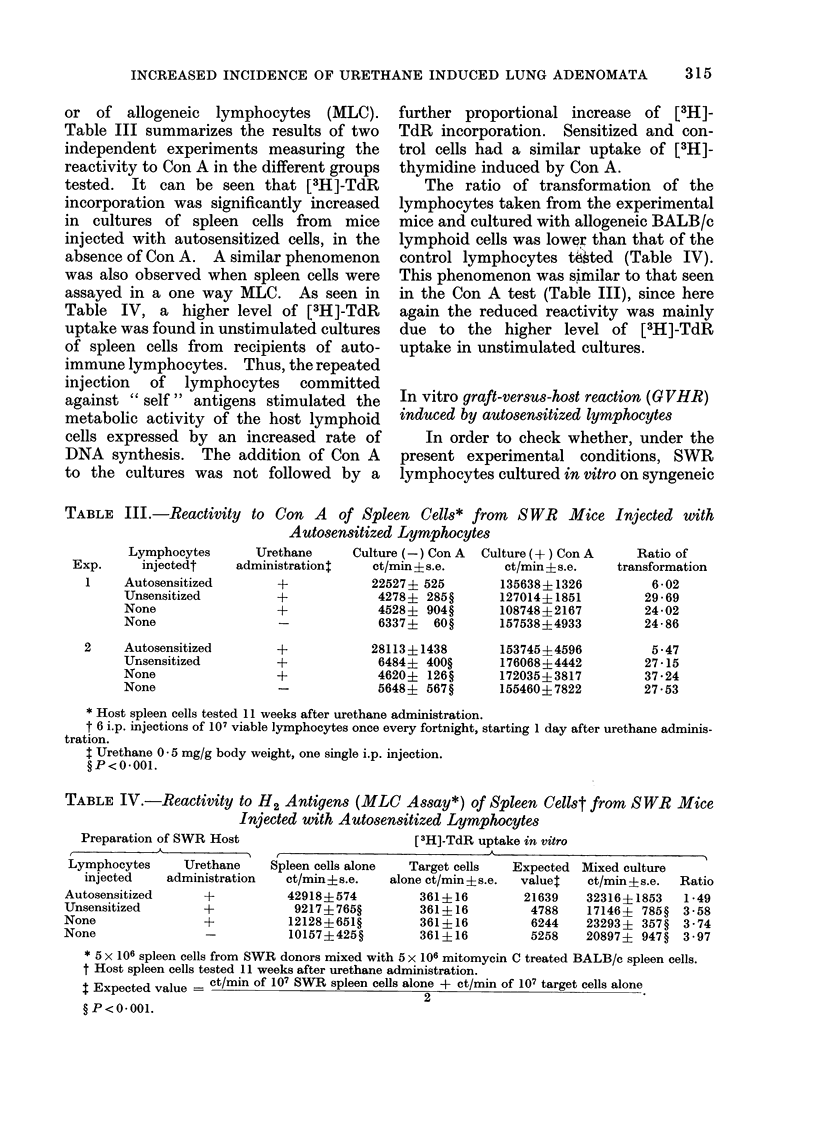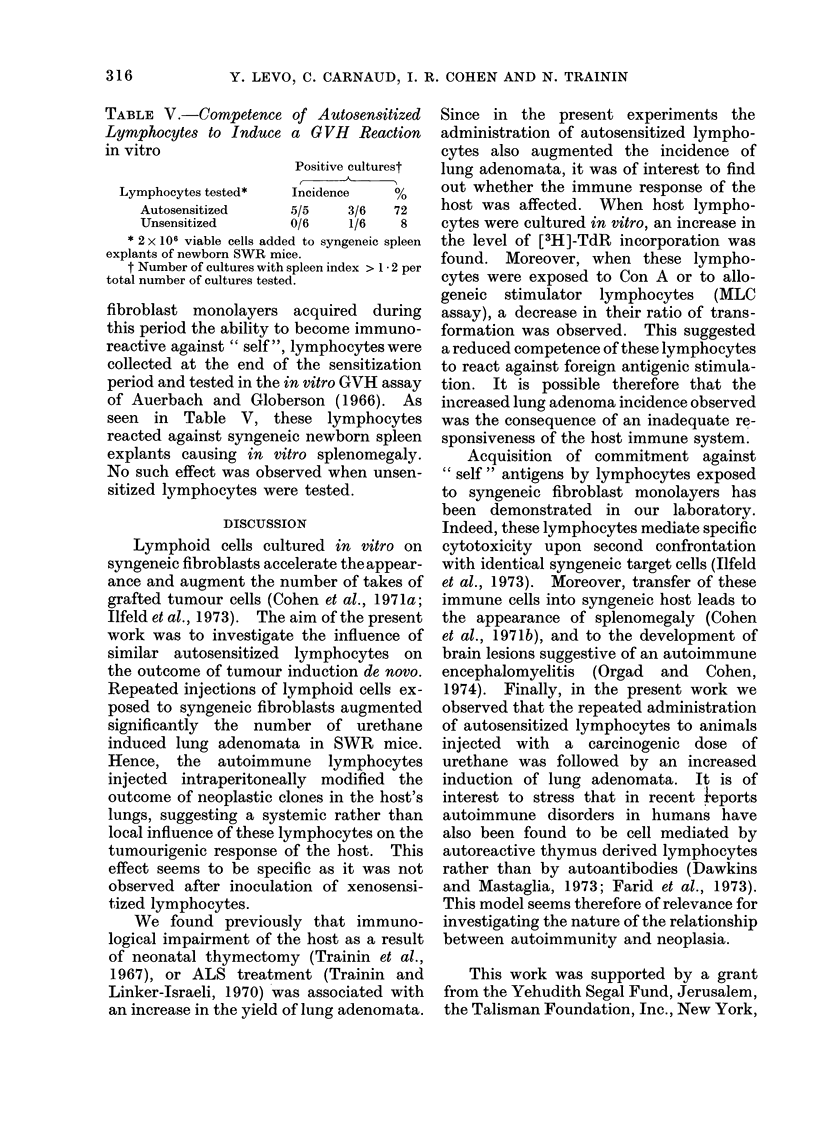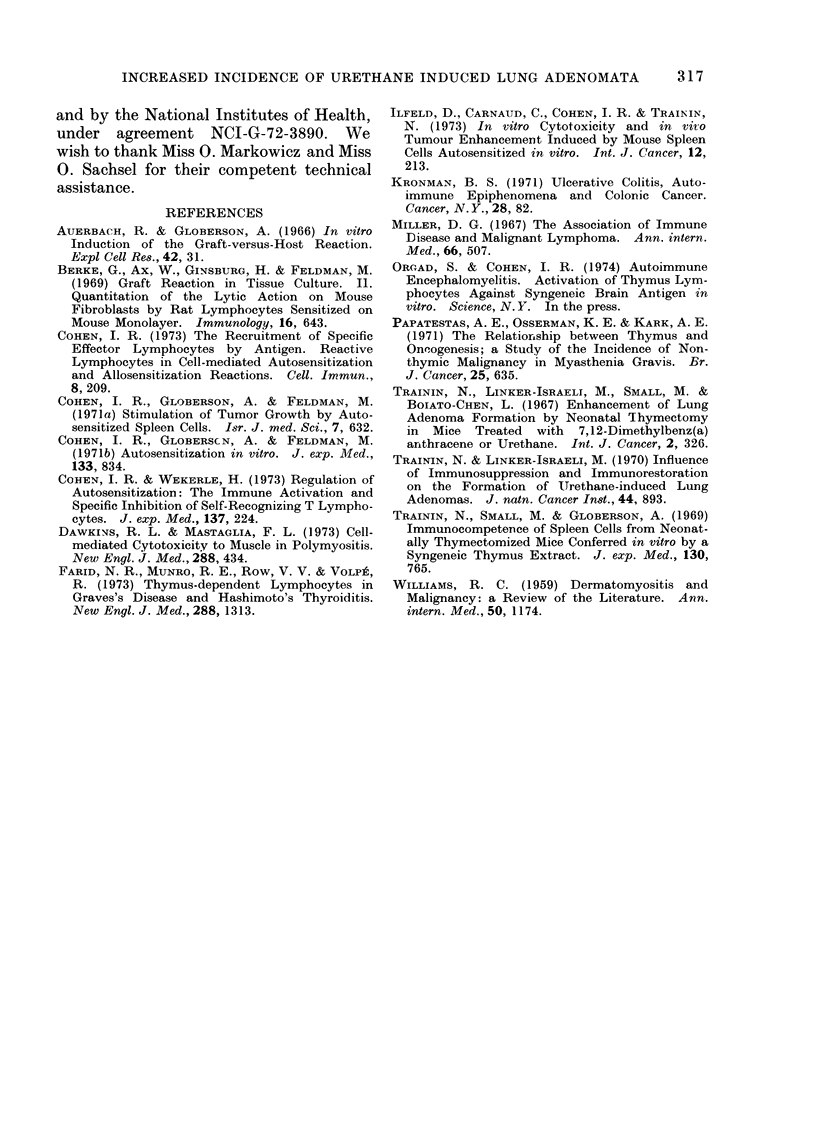# Increased Incidence of Urethane Induced Lung Adenomata by Autosensitized Lymphocytes

**DOI:** 10.1038/bjc.1974.72

**Published:** 1974-04

**Authors:** Y. Levo, C. Carnaud, I. R. Cohen, N. Trainin

## Abstract

The present work investigates the influence of autosensitized lymphocytes on the carcinogenic response of the host. Urethane treated SWR mice received 6 fortnightly injections of lymphocytes sensitized *in vitro* against syngeneic fibroblasts. An increased incidence of lung adenomata was found in these mice compared with controls injected with unsensitized lymphoid cells or with lymphoid cells sensitized against unrelated transplantation antigens. Autosensitized lymphocytes also modified the response of host lymphoid cells to concanavalin A or to stimulation in a mixed lymphocyte culture assay. These results indicate that autoimmune lymphocytes may increase susceptibility of a host to the induction of tumours.


Br. J. Cancer (1974) 29, 312

### INCREASED INCIDENCE OF URETHANE INDUCED LUNG ADENOMATA BY AUTOSENSITIZED LYMPHOCYTES

Y. LEVO,* C. CARNAUD, I. R. COHEN AND N. TRAININ†

*From the Department of Cell Biology, The Weizmann Institute of Science, Rehovot, Israel*

Received 4 December 1973. Accepted 10 January 1974

**Summary.—The present work investigates the influence of autosensitized lymphocytes on the carcinogenic response of the host. Urethane treated SWR mice received 6 fortnightly injections of lymphocytes sensitized *in vitro* against syngeneic fibroblasts. An increased incidence of lung adenomata was found in these mice compared with controls injected with unsensitized lymphoid cells or with lymphoid cells sensitized against unrelated transplantation antigens. Autosensitized lymphocytes also modified the response of host lymphoid cells to concanavalin A or to stimulation in a mixed lymphocyte culture assay. These results indicate that autoimmune lymphocytes may increase susceptibility of a host to the induction of tumours.**

SEVERAL observations suggest that autoimmune diseases are associated with an increased incidence of malignancies. A higher probability of incidence of both lymphoreticular and solid tumours has been reported in surveys of patients afflicted with myasthenia gravis, lupus erythematosus, ulcerative colitis and other diseases considered to have an autoimmune component (Williams, 1959; Miller, 1967; Kronman, 1971; Papatestas, Osserman and Kark, 1971). A reproducible model of an autoimmune disease in animals is needed for further investigation of the mechanisms underlying these observations.

It has been previously shown in our laboratory that T lymphocytes cultured *in vitro* on syngeneic cell monolayers become sensitized against self antigens (Cohen and Wekerle, 1973). These autosensitized lymphocytes were found to mediate specific cytotoxicity against syngeneic target cells (Ilfeld *et al.*, 1973), and to produce splenomegaly or enlargement of draining lymph nodes after injection into syngeneic recipient animals (Cohen, Globerson and Feldman, 1971*a*; Cohen, 1973). Preliminary studies were then undertaken to test the effects of autosensitized lymphocytes on the growth of tumour cells. Mouse lymphocytes were autosensitized against syngeneic fibroblasts, mixed together with syngeneic tumour cells and injected into recipient mice. We found that autosensitized lymphocytes enhanced the growth of tumour cells simultaneously inoculated (Cohen *et al.*, 1971*b*; Ilfeld *et al.*, 1973). These observations did not, however, answer the question whether autoimmunity has any effect upon the induction of tumours *de novo*.

It was therefore decided to test whether the adoptive transfer of lymphocytes sensitized *in vitro* against syngeneic fibroblasts would modify the carcinogenic response of mice submitted to urethane treatment. We found in the present experiments that SWR mice repeatedly injected with autosensitized lymphocytes displayed a higher incidence of lung adenomata, when compared with controls. In parallel, the immunological status of these animals was evaluated by measuring the reactivity of their spleen cells to

* Department of Internal Medicine B, Beilinson Hospital, Petah Tikva, Israel.
† Established Investigator of the Chief Scientist's Bureau, Ministry of Health, Israel.

concanavalin A and to allogeneic lymphoid cells in a mixed lymphocyte culture (MLC) assay.

### MATERIALS AND METHODS

*Animals.*—One and a half month-old inbred female SWR mice were used throughout the experiments. BALB/c mice and Lewis rats were used in some of the experiments as a source of cells. The animals were supplied by the Animal Breeding Center of The Weizmann Institute of Science. Homozygosity was tested routinely by skin grafting.

*Preparation of spleen cells.*—Spleens were removed surgically, the tissues placed in cold Dulbecco's modified Eagle's medium (EM) and pressed through a fine stainless steel mesh. The cell suspensions obtained were washed and centrifuged twice at 600 *g* for 5 min, counted in a haemacytometer and resuspended in EM. When used for *in vitro* sensitization the EM was supplemented with 15% foetal calf serum (FCS, Grand Island Biological Co., Berkeley, California).

*Fibroblast monolayers.*—Fibroblast monolayers were obtained from mouse or rat embryos at 13–16 days of gestation, as previously described (Berke, Ginsburg and Feldman, 1969) and grown in 60 mm plastic petri dishes at a concentration of $2 \times 10^6$ cells in 4 ml of Waymouth's medium plus 5% calf serum. The fibroblasts were used after one transfer, which was generally performed 1 week after primary culture.

In vitro *sensitization of spleen cells.*—Fibroblast monolayers were irradiated (2000 rad from a $^{60}$Cobalt machine, Atomic Energy Ltd., Ottawa, Canada, gamma beam 150 Å, dose rate of 1000 rad/min). A few hours later, $40 \times 10^6$ viable nucleated spleen cells in 4 ml of EM+15% FCS were poured into petri dishes containing the sensitizing monolayer, and incubated at 37°C in humidified air plus 5% $CO_2$. After 24 h of incubation, the medium was replaced by fresh EM. On the fourth day the sensitized lymphoid cells were recovered from the monolayer by careful pipetting, washed twice, counted in a haemacytometer in the presence of trypan blue and resuspended in EM at the desired concentration.

*Induction of lung adenomata in SWR mice.*—SWR mice were injected once, i.p., with a 5% aqueous solution of urethane (British Drug Houses, Ltd., Poole, England), on the basis of 0·5 mg/g body weight. Eleven–12 weeks after urethane administration the mice were killed, their lungs examined with a low-power dissecting microscope and the number of lung adenomata recorded, as described previously (Trainin *et al.*, 1967).

*Lymphocyte transformation induced by Concanavalin A (Con A).*—Five million nucleated spleen cells in 1 ml of EM+10% FCS were incubated for 72 h in the presence of 2 μg of Con A (Miles-Yeda, Rehovot, Israel). Two hours before the end of the reaction, 2 μCi of [$^3$H]-thymidine ([$^3$H]-TdR) (Radiochemical Centre, Amersham, England) were added to the cultures, which were then gently shaken in a 37°C water bath. The cells were then poured on to fibreglass filters (Wathman, England) and rinsed successively with saline, a cold solution of 5% TCA and absolute ethanol. The filters were dried, immersed into PPO-POPOP toluene and counted in a Packard scintillation counter

*Mixed lymphocyte reaction.*—Five million nucleated spleen cells taken from the various groups of SWR mice and suspended in 1 ml of EM+10% FCS were mixed together with 1 ml containing $5 \times 10^6$ mitomycin C (Sigma) treated spleen cells from normal BALB/c mice. Treatment with mitomycin C was performed by incubation of $100 \times 10^6$ spleen cells in the presence of 0·5 g mitomycin C in 10 ml EM for 30 min in a 37°C shaking bath. Afterwards, the cells were washed 5 times with EM and resuspended in EM+10% FCS.

The cell mixtures were incubated for 4 days. Radioactive thymidine pulse, extraction and counting were carried out as described for the Con A assay.

In vitro *assay of graft versus host reaction.*—The assay was performed as described by Auerbach and Globerson (1966). Cultures of spleen fragments from newborn SWR mice were exposed to $2 \times 10^6$ autosensitized spleen cells or to $2 \times 10^6$ unsensitized spleen cells. Cultures were scored after 4 days. The relative enlargement of the spleen fragment in each culture was compared with a paired explant of spleen exposed to control spleen cells. The calculated area of each test spleen fragment divided by the corresponding area of its paired reference fragment provided a numerical index of splenomegaly. Cultures were considered reactive when the index of splenomegaly obtained was 1·2 or more (Trainin, Small and Globerson, 1969).

### RESULTS

### *Influence of repeated injections of autosensitized lymphocytes on the incidence of urethane induced lung adenomata*

SWR mice were injected i.p. every fortnight with *in vitro* autosensitized lymphocytes. The animals received a total of 6 injections of $10^7$ viable lymphoid cells each. Control groups included uninjected mice, and mice injected with unsensitized lymphoid cells according to the schedule described above. Experimental and control groups of animals received 0·5 mg/g body weight urethane one day before the first lymphoid cell injection. The weight of the animals was recorded weekly and no differences were observed between experimental and control mice. Eleven weeks after urethane administration the animals were killed and the lungs examined for the presence of adenomata. As seen in Table I, the incidence of lung adenomata was significantly higher in the group of mice treated with autosensitized spleen cells (4·1) compared with recipients of normal lymphocytes (2·1), or with recipients of urethane only (2·6). Thus, repeated injections of autosensitized lymphocytes increase the incidence of urethane induced lung adenomata.

Experiments were then made to see whether the enhancement of lung adenoma formation was specific to the injection of lymphocytes sensitized against " self ". The previous experimental model was used with the addition of a further control group consisting of mice injected with syngeneic lymphocytes xenosensitized against rat fibroblasts. Table II shows that xenosensitized lymphocytes did not affect the incidence of lung adenomata.

### *T cell reactivity of spleen cells from mice submitted to repeat inoculation of autoimmune lymphocytes*

We have reported previously that an impairment of thymus function was followed by a higher carcinogenic response to urethane in mice (Trainin *et al.*, 1967; Trainin and Linker-Israeli, 1970). The effects of the injection of autosensitized lymphocytes upon the T cell competence of the recipients in our experiments was examined. Spleen cells from injected or uninjected mice were tested for *in vitro* transformation in the presence of Con A

TABLE I.—*Influence of Autosensitized Lymphocytes on the Incidence of Urethane* Induced Lung Adenomata in SWR Mice*

| Lymphocytes injected† | No. of injections | No. of animals | No. of adenomata per animal Mean±s.e. | *P* value‡ |
|---|---|---|---|---|
| Autosensitized | 6 | 14 | 4·14±0·59 | |
| Unsensitized | 6 | 16 | 2·06±0·21 | <0·002 |
| None | — | 14 | 2·57±0·42 | <0·025 |

* Urethane 0·5 mg/g body weight, one single i.p. injection.
† 6 i.p. injections of $10^7$ viable cells once every fortnight, starting 1 day after urethane administration.
‡ Calculated according to Student's *t* test.

TABLE II.—*Influence of Autosensitized Versus Xenosensitized* Lymphocytes on the Incidence of Urethane Induced Lung Adenomata in SWR Mice*

| Lymphocytes injected | No. of injections | No. of animals | No. of adenomata per animal Mean±s.e. | *P* value |
|---|---|---|---|---|
| Autosensitized | 6 | 12 | 6·83±1·12 | — |
| Xenosensitized | 6 | 15 | 3·07±0·57 | <0·005 |
| Unsensitized | 6 | 15 | 2·27±0·40 | <0·001 |
| None | — | 14 | 2·93±0·49 | <0·005 |

* SWR spleen cells sensitized *in vitro* on monolayers of rat BN fibroblasts.

or of allogeneic lymphocytes (MLC). Table III summarizes the results of two independent experiments measuring the reactivity to Con A in the different groups tested. It can be seen that [$^3$H]-TdR incorporation was significantly increased in cultures of spleen cells from mice injected with autosensitized cells, in the absence of Con A. A similar phenomenon was also observed when spleen cells were assayed in a one way MLC. As seen in Table IV, a higher level of [$^3$H]-TdR uptake was found in unstimulated cultures of spleen cells from recipients of autoimmune lymphocytes. Thus, the repeated injection of lymphocytes committed against "self" antigens stimulated the metabolic activity of the host lymphoid cells expressed by an increased rate of DNA synthesis. The addition of Con A to the cultures was not followed by a further proportional increase of [$^3$H]-TdR incorporation. Sensitized and control cells had a similar uptake of [$^3$H]-thymidine induced by Con A.

The ratio of transformation of the lymphocytes taken from the experimental mice and cultured with allogeneic BALB/c lymphoid cells was lower than that of the control lymphocytes tested (Table IV). This phenomenon was similar to that seen in the Con A test (Table III), since here again the reduced reactivity was mainly due to the higher level of [$^3$H]-TdR uptake in unstimulated cultures.

*In vitro graft-versus-host reaction (GVHR) induced by autosensitized lymphocytes*

In order to check whether, under the present experimental conditions, SWR lymphocytes cultured *in vitro* on syngeneic

TABLE III.—*Reactivity to Con A of Spleen Cells* from SWR Mice Injected with Autosensitized Lymphocytes*

| Exp. | Lymphocytes injected† | Urethane administration‡ | Culture (−) Con A ct/min ± s.e. | Culture (+) Con A ct/min ± s.e. | Ratio of transformation |
|---|---|---|---|---|---|
| 1 | Autosensitized | + | 22527 ± 525 | 135638 ± 1326 | 6·02 |
| | Unsensitized | + | 4278 ± 285§ | 127014 ± 1851 | 29·69 |
| | None | + | 4528 ± 904§ | 108748 ± 2167 | 24·02 |
| | None | − | 6337 ± 60§ | 157538 ± 4933 | 24·86 |
| 2 | Autosensitized | + | 28113 ± 1438 | 153745 ± 4596 | 5·47 |
| | Unsensitized | + | 6484 ± 400§ | 176068 ± 4442 | 27·15 |
| | None | + | 4620 ± 126§ | 172035 ± 3817 | 37·24 |
| | None | − | 5648 ± 567§ | 155460 ± 7822 | 27·53 |

* Host spleen cells tested 11 weeks after urethane administration.
† 6 i.p. injections of $10^7$ viable lymphocytes once every fortnight, starting 1 day after urethane administration.
‡ Urethane 0·5 mg/g body weight, one single i.p. injection.
§ $P < 0{\cdot}001$.

TABLE IV.—*Reactivity to $H_2$ Antigens (MLC Assay*) of Spleen Cells† from SWR Mice Injected with Autosensitized Lymphocytes*

| Preparation of SWR Host | | [$^3$H]-TdR uptake *in vitro* | | | | |
|---|---|---|---|---|---|---|
| Lymphocytes injected | Urethane administration | Spleen cells alone ct/min ± s.e. | Target cells alone ct/min ± s.e. | Expected value‡ | Mixed culture ct/min ± s.e. | Ratio |
| Autosensitized | + | 42918 ± 574 | 361 ± 16 | 21639 | 32316 ± 1853 | 1·49 |
| Unsensitized | + | 9217 ± 765§ | 361 ± 16 | 4788 | 17146 ± 785§ | 3·58 |
| None | + | 12128 ± 651§ | 361 ± 16 | 6244 | 23293 ± 357§ | 3·74 |
| None | − | 10157 ± 425§ | 361 ± 16 | 5258 | 20897 ± 947§ | 3·97 |

* $5 \times 10^6$ spleen cells from SWR donors mixed with $5 \times 10^6$ mitomycin C treated BALB/c spleen cells.
† Host spleen cells tested 11 weeks after urethane administration.
‡ Expected value $= \frac{\text{ct/min of } 10^7 \text{ SWR spleen cells alone} + \text{ct/min of } 10^7 \text{ target cells alone}}{2}$.
§ $P < 0{\cdot}001$.

TABLE V.—*Competence of Autosensitized Lymphocytes to Induce a GVH Reaction* in vitro

| Lymphocytes tested* | Positive cultures† | | |
|---|---|---|---|
| | Incidence | | % |
| Autosensitized | 5/5 | 3/6 | 72 |
| Unsensitized | 0/6 | 1/6 | 8 |

* $2 \times 10^6$ viable cells added to syngeneic spleen explants of newborn SWR mice.

† Number of cultures with spleen index > 1·2 per total number of cultures tested.

fibroblast monolayers acquired during this period the ability to become immunoreactive against " self ", lymphocytes were collected at the end of the sensitization period and tested in the *in vitro* GVH assay of Auerbach and Globerson (1966). As seen in Table V, these lymphocytes reacted against syngeneic newborn spleen explants causing *in vitro* splenomegaly. No such effect was observed when unsensitized lymphocytes were tested.

### DISCUSSION

Lymphoid cells cultured *in vitro* on syngeneic fibroblasts accelerate the appearance and augment the number of takes of grafted tumour cells (Cohen *et al.*, 1971*a*; Ilfeld *et al.*, 1973). The aim of the present work was to investigate the influence of similar autosensitized lymphocytes on the outcome of tumour induction *de novo*. Repeated injections of lymphoid cells exposed to syngeneic fibroblasts augmented significantly the number of urethane induced lung adenomata in SWR mice. Hence, the autoimmune lymphocytes injected intraperitoneally modified the outcome of neoplastic clones in the host's lungs, suggesting a systemic rather than local influence of these lymphocytes on the tumourigenic response of the host. This effect seems to be specific as it was not observed after inoculation of xenosensitized lymphocytes.

We found previously that immunological impairment of the host as a result of neonatal thymectomy (Trainin *et al.*, 1967), or ALS treatment (Trainin and Linker-Israeli, 1970) was associated with an increase in the yield of lung adenomata. Since in the present experiments the administration of autosensitized lymphocytes also augmented the incidence of lung adenomata, it was of interest to find out whether the immune response of the host was affected. When host lymphocytes were cultured *in vitro*, an increase in the level of [$^3$H]-TdR incorporation was found. Moreover, when these lymphocytes were exposed to Con A or to allogeneic stimulator lymphocytes (MLC assay), a decrease in their ratio of transformation was observed. This suggested a reduced competence of these lymphocytes to react against foreign antigenic stimulation. It is possible therefore that the increased lung adenoma incidence observed was the consequence of an inadequate responsiveness of the host immune system.

Acquisition of commitment against " self " antigens by lymphocytes exposed to syngeneic fibroblast monolayers has been demonstrated in our laboratory. Indeed, these lymphocytes mediate specific cytotoxicity upon second confrontation with identical syngeneic target cells (Ilfeld *et al.*, 1973). Moreover, transfer of these immune cells into syngeneic host leads to the appearance of splenomegaly (Cohen *et al.*, 1971*b*), and to the development of brain lesions suggestive of an autoimmune encephalomyelitis (Orgad and Cohen, 1974). Finally, in the present work we observed that the repeated administration of autosensitized lymphocytes to animals injected with a carcinogenic dose of urethane was followed by an increased induction of lung adenomata. It is of interest to stress that in recent reports autoimmune disorders in humans have also been found to be cell mediated by autoreactive thymus derived lymphocytes rather than by autoantibodies (Dawkins and Mastaglia, 1973; Farid *et al.*, 1973). This model seems therefore of relevance for investigating the nature of the relationship between autoimmunity and neoplasia.

This work was supported by a grant from the Yehudith Segal Fund, Jerusalem, the Talisman Foundation, Inc., New York, and by the National Institutes of Health, under agreement NCI-G-72-3890. We wish to thank Miss O. Markowicz and Miss O. Sachsel for their competent technical assistance.

### REFERENCES

AUERBACH, R. & GLOBERSON, A. (1966) *In vitro* Induction of the Graft-versus-Host Reaction. *Expl Cell Res.*, **42**, 31.

BERKE, G., AX, W., GINSBURG, H. & FELDMAN, M. (1969) Graft Reaction in Tissue Culture. II. Quantitation of the Lytic Action on Mouse Fibroblasts by Rat Lymphocytes Sensitized on Mouse Monolayer. *Immunology*, **16**, 643.

COHEN, I. R. (1973) The Recruitment of Specific Effector Lymphocytes by Antigen. Reactive Lymphocytes in Cell-mediated Autosensitization and Allosensitization Reactions. *Cell. Immun.*, **8**, 209.

COHEN, I. R., GLOBERSON, A. & FELDMAN, M. (1971*a*) Stimulation of Tumor Growth by Autosensitized Spleen Cells. *Isr. J. med. Sci.*, **7**, 632.

COHEN, I. R., GLOBERSON, A. & FELDMAN, M. (1971*b*) Autosensitization *in vitro*. *J. exp. Med.*, **133**, 834.

COHEN, I. R. & WEKERLE, H. (1973) Regulation of Autosensitization: The Immune Activation and Specific Inhibition of Self-Recognizing T Lymphocytes. *J. exp. Med.*, **137**, 224.

DAWKINS, R. L. & MASTAGLIA, F. L. (1973) Cell-mediated Cytotoxicity to Muscle in Polymyositis. *New Engl. J. Med.*, **288**, 434.

FARID, N. R., MUNRO, R. E., ROW, V. V. & VOLPÉ, R. (1973) Thymus-dependent Lymphocytes in Graves's Disease and Hashimoto's Thyroiditis. *New Engl. J. Med.*, **288**, 1313.

ILFELD, D., CARNAUD, C., COHEN, I. R. & TRAININ, N. (1973) *In vitro* Cytotoxicity and *in vivo* Tumour Enhancement Induced by Mouse Spleen Cells Autosensitized *in vitro*. *Int. J. Cancer*, **12**, 213.

KRONMAN, B. S. (1971) Ulcerative Colitis, Autoimmune Epiphenomena and Colonic Cancer. *Cancer, N.Y.*, **28**, 82.

MILLER, D. G. (1967) The Association of Immune Disease and Malignant Lymphoma. *Ann. intern. Med.*, **66**, 507.

ORGAD, S. & COHEN, I. R. (1974) Autoimmune Encephalomyelitis. Activation of Thymus Lymphocytes Against Syngeneic Brain Antigen *in vitro*. *Science, N.Y.* In the press.

PAPATESTAS, A. E., OSSERMAN, K. E. & KARK, A. E. (1971) The Relationship between Thymus and Oncogenesis; a Study of the Incidence of Nonthymic Malignancy in Myasthenia Gravis. *Br. J. Cancer*, **25**, 635.

TRAININ, N., LINKER-ISRAELI, M., SMALL, M. & BOIATO-CHEN, L. (1967) Enhancement of Lung Adenoma Formation by Neonatal Thymectomy in Mice Treated with 7,12-Dimethylbenz(a) anthracene or Urethane. *Int. J. Cancer*, **2**, 326.

TRAININ, N. & LINKER-ISRAELI, M. (1970) Influence of Immunosuppression and Immunorestoration on the Formation of Urethane-induced Lung Adenomas. *J. natn. Cancer Inst.*, **44**, 893.

TRAININ, N., SMALL, M. & GLOBERSON, A. (1969) Immunocompetence of Spleen Cells from Neonatally Thymectomized Mice Conferred *in vitro* by a Syngeneic Thymus Extract. *J. exp. Med.*, **130**, 765.

WILLIAMS, R. C. (1959) Dermatomyositis and Malignancy: a Review of the Literature. *Ann. intern. Med.*, **50**, 1174.